# Bioinformatics analysis of the prognostic value of NEK8 and its effects on immune cell infiltration in glioma

**DOI:** 10.1111/jcmm.16831

**Published:** 2021-08-10

**Authors:** Meng Xiao, Chaoyang Du, Chuanbo Zhang, Xinzhong Zhang, Shaomin Li, Dainan Zhang, Wang Jia

**Affiliations:** ^1^ Henan Key Laboratory of Neurorestoratology The First Affiliated Hospital of Xinxiang Medical University Weihui China; ^2^ Beijing Neurosurgical Institute Capital Medical University Beijing China; ^3^ Department of Neurosurgery Beijing Tiantan Hospital Capital Medical University Beijing China; ^4^ Chinese Glioma Genome Atlas Network (CGGA) Beijing China; ^5^ Department of Neurology Ann Romney Center for Neurologic Diseases Brigham and Women’s Hospital and Harvard Medical School Boston MA USA

**Keywords:** biomarker, DNA damage response, glioma, NEK8, prognosis, tumour microenvironment

## Abstract

Glioma is the most common malignancy of the nervous system with high rates of recurrence and mortality, even after surgery. The 5‐year survival rate is only about 5%. NEK8 is involved in multiple biological processes in a variety of cancers; however, its role in glioma is still not clear. In the current study, we evaluated the prognostic value of NEK8, as well as its role in the pathogenesis of glioma. Using a bioinformatics approach and RNA‐seq data from public databases, we found that NEK8 expression is elevated in glioma tissues; we further verified this result by RT‐PCR, Western blotting and immunochemistry using clinical samples. Functional enrichment analyses of genes with correlated expression indicated that elevated NEK8 expression is associated with increased immune cell infiltration in glioma and may affect the tumour microenvironment via the regulation of DNA damage/repair. Survival analyses revealed that high levels of NEK8 are associated with a poorer prognosis; higher WHO grade, IDH status, 1p/19q codeletion, age and NEK8 were identified as an independent prognostic factor. These findings support the crucial role of NEK8 in the progression of glioma via effects on immune cell infiltration and suggest that it is a new prognostic biomarker.

## 1 INTRODUCTION

Glioma is the most lethal and common type of malignancy found in the nervous system and presents a significant burden to the public health system worldwide.^1^ Due to the difficulty of complete surgical resection, high recurrence rates after surgery lead to poor outcomes and high mortality rates. Surgical resection followed by radiation with concomitant and adjuvant temozolomide has improved outcomes in some patient subsets.^2^, ^3^ However, overall outcomes are still poor, with a median survival time of less than 15 months.^4^ Thus, detailed analyses of the molecular mechanisms are needed for the identification of new prognostic markers.

NEK family members, Nek1 to Nek11,^5^ encode serine/threonine‐specific protein kinases that are widely expressed in cilia, centrosomes, nuclei, cytoplasm and mitochondria. As cell cycle kinases, NEKs are related to the mitotic regulator ‘never in mitosis, gene A’ (NIMA)^6^, ^7^ and regulate the cell cycle progression from the G2 to M phase.^8^, ^9^ NEKs are associated with multiple cancers.^10^, ^11^ Furthermore, their involvement in specific aspects of microtubule function and the DNA damage checkpoint, a key target pathway for cancer drugs, has led to considerable interest in mitotic enzymes as candidate cancer drug targets.

NEK2 is overexpressed in many human tumours. Its depletion prevents centrosome separation, blocks mitosis and increases apoptosis.^12^ It has been identified as a therapeutic target in breast cancer,^13^ cholangiocarcinoma and colorectal cancer,^14^ to name a few. Similarly, NEK3 overexpression in human breast cancer affects prolactin receptor signalling and upregulates Vav2 phosphorylation.^15^ Nek6 has also been identified as a tumorigenesis protein and potential therapeutic target in cancer. For instance, the activation of Nek6 facilitates anchorage‐independent growth. Its depletion leads to cancer cell death,^16^ and its overexpression has been reported to suppress p53‐dependent cellular senescence.^17^

NEK8 is of particular interest, owing to the discovery of specific mutations that cause polycystic kidney disease in zebrafish and mice.^18^ NEK8 is a 692 amino acid protein with a molecular weight of 75 kDa.^19^ The protein consists of an N‐terminal catalytic domain and a C‐terminal domain, which are typical characteristics of serine/threonine kinases, including other mitotic kinases.^6^ Missense mutations in NEK8 may induce defects in DNA repair and increased apoptosis.^20^ These findings indicate that the dysfunction of NEK8 may contribute to tumorigenesis.^21^ For example, missense mutations in NEK8 are underlying driver mutations in pancreatic cancer.^22^ Bowers et al.^23^ found that NEK8 expression is elevated in human breast cancer. However, the fundamental mechanism by which NEK8 contributes to glioma is still poorly understood.

In this study, we investigated NEK8 expression and its prognostic value in glioma using clinical samples and data from The Cancer Genome Atlas (TCGA) and Chinese Glioma Genome Atlas (CGGA). Furthermore, we assessed correlations between clinical‐pathologic features and the expression of NEK8. Finally, we evaluated the biological functions and pathways associated with NEK8. Our results provide potential therapeutic targets in glioma, as well as novel insights into the molecular mechanisms underlying the effects of NEK8.

## 2 MATERIALS AND METHODS

### 2.1 Patients and specimens

Patient specimens for PCR, Western blotting, immunohistochemical (IHC) staining and flow cytometry were collected at the Beijing Tiantan Hospital from August 2019 to June 2021. Three non‐tumour brain samples from traumatic brain injury internal decompression and nine tumour samples (three grade II, three grade III and three grade IV cases) were acquired for the PCR assay. Six non‐tumour samples and four WHO II, III and IV glioma samples (different from the samples used for PCR) were used for Western blotting. Besides, the above clinical specimens and other samples were collected for the IHC staining and flow cytometry. The clinical information of patients was shown in Table S1. Two neuropathologists confirmed the histological diagnosis of the specimens according to the classification guidelines of the 2016 World Health Organization (WHO). Informed consent was obtained from all patients, and the study was approved by the Ethics Committee of Beijing Tiantan Hospital, Capital Medical University (KY 2018–052–01).

### 2.2 RNA isolation and quantitative RT‐PCR

Total RNA was isolated from clinical tissues using an RNA Kit (Omega) based on the manufacturer's protocol. RNA was reverse transcribed into cDNA via NovoScript II Reverse Transcriptase (Novoprotein). Quantitative PCR was performed using a Biosystem thermal cycler (Life Technologies, Singapore) and NovoStart^®^ SYBR qPCR SuperMix Assay (Novoprotein). The relative mRNA expression levels of NEK8 and GAPDH were calculated using the 2^−ΔΔCt^ method. The primers were as follows: NEK8 forward 5′‐ATGGCAGCCTCACTGACATCAG‐3′ and reverse 5′‐CCAGGCAAATAGTTCTCGCTCAG‐3′ and GAPDH forward 5′‐CTCCTCCACCTTTGACGCTG‐3′ and reverse 5′‐TCCTCTTGTGCTCTTGCTGG‐3′.

### 2.3 Western blot analysis

The clinical samples were lysed with RIPA lysate buffer. The supernatants were collected after centrifugation and then boiled for 5 minutes with loading buffer (40% glycerol, 100‐mM Tris, bromophenol blue) at a ratio of 25:100. Proteins were separated using sodium dodecyl sulphate‐polyacrylamide gel electrophoresis (SDS‐PAGE) and transferred to polyvinylidene fluoride (PVDF) membranes. The membranes were blocked in 5% non‐fat milk in TBS‐T for 1 hour at room temperature and then incubated with anti‐NEK8 (1:2000) and anti‐GAPDH (1:5000) antibodies (Abcam) overnight at 4°C. Subsequently, the membranes were washed and incubated with secondary anti‐rabbit antibodies (1:5000) coupled to horseradish peroxidase for 1 hour at room temperature. Finally, the protein expression levels of samples were detected using a chemiluminescence (ECL) system.

### 2.4 Immunohistochemistry

The sections were deparaffinized with xylene and rehydrated with gradient ethanol, and antigen retrieval was carried out in a microwave oven with citric acid. Endogenous peroxidase activity was blocked using 3% H_2_O_2_. The sections were incubated in 10% normal goat serum to block the non‐specific binding of the antibody and were incubated with anti‐NEK8 (dilution 1:100; Abcam) as the primary antibody overnight at 4°C. The sections were subsequently incubated for 30 minutes at room temperature with the secondary antibody conjugated with horseradish peroxidase, immersed in diaminobenzidine(DAB) and counterstained with haematoxylin for 2 minutes.

### 2.5 Flow cytometry

Clinical tissues were rinsed in PBS, minced into fine pieces and digested at 37°C for 1 hour in 0.5 g/L collagenase (Sigma‐Aldrich), then incubated in 10% foetal bovine serum (FBS) (Gibco). Digests were filtered through a 75‐μm mesh for single‐cell isolation. After a centrifugation at 1500 rpm for 5 minutes, the cell pellet was collected in the bottom. After counting, cells were washed twice with PBS. Then, antibodies against CD45‐APC‐750, CD16‐ECD, CD56‐PE, CD3‐PE‐Cy5.5, CD4‐PE‐Cy7, CD194‐APC (BioLegend) were added and incubated in the dark for 30 minutes on ice. Single cells were washed twice in PBS before analysis on a Beckman Coulter CytoFLEX flow cytometer. Analysis of flow cytometry results was performed using FlowJo software.

### 2.6 Public databases

RNA‐seq data were downloaded from TCGA and GTEx using UCSC XENA (https://xenabrowser.net/datapages/). These data were uniformly transformed into TPM (transcripts per million reads) by the Toil process [22] for comparative analyses. The Wilcoxon rank‐sum test was used to compare NEK8 levels in normal samples from GTEx combined with TCGA and tumour samples obtained from TCGA. Expression profiles (HT Seq‐Counts) were compared between high and low NEK8 expression groups using the median value as the cut‐off to identify differentially expressed genes (DEGs) using the DESeq2 (3.8) package [23]. A volcano plot and heat map were generated for visualization. RNA‐seq data were also obtained from CGGA (http://www.cgga.org.cn/). The Wilcoxon rank‐sum test and Wilcoxon signed‐rank test were used to compare NEK8 expression levels between tumour samples and control samples. The Kruskal‐Wallis test, Wilcoxon signed‐rank test and Spearman's correlation coefficients were used to assess the correlations between clinical‐pathologic features and the expression of NEK8. The Pearson chi‐square test was used to analyse the direct correlation between high and low NEK8 groups and the grade of clinicopathologic factors (Fisher's exact test was used when needed). All statistical analyses and the generation of plots were performed using R (v3.6.2).

### 2.7 Prognostic analysis and nomogram

To evaluate prognostic factors, the Kaplan‐Meier method was used to construct survival curves. Additionally, relationships between survival and clinical factors, including gender, age, race, WHO grade, IDH status, 1p/19q codeletion, primary therapy outcome, EGFR status, PIK3CA status and NEK8 level, were evaluated. Univariate Cox regression analyses were performed. Then, significant variables from the univariate analyses (*p* < 0.1) were included in a multivariate analysis to confirm independent predictors. To precisely predict the 1‐year, 3‐year and 5‐year survival probabilities, a nomogram was constructed based on the results of the multivariate analysis. Furthermore, we compared the predictive accuracy of the nomogram with respective prognostic factors based on the C‐index and receiver operating characteristic (ROC) analyses. Finally, we constructed a calibration curve to evaluate the predictive value for overall survival (OS), progression‐free interval (PFI) and disease‐specific survival (DSS).

### 2.8 Functional enrichment analyses

Functional enrichment analyses of DEGs, including gene ontology (GO) and Kyoto Encyclopedia of Genes and Genomes (KEGG) pathway analyses, were performed using clusterProfiler.^24^ Immune infiltration associated with NEK8 was evaluated via single‐sample gene set enrichment analysis (ssGSEA) using the GSVA package (http://www.bioconductor.org/packages/release/bioc/html/GSVA.html). The relative levels of each tumour‐infiltrating immunocyte were quantified according to the signature genes of 24 immune cell types.^25^ Spearman's correlation coefficients were determined to evaluate the correlation between *NEK8* and levels of immune cell infiltration. The Search Tool for the Retrieval of Interacting Genes (STRING) (http://string‐db.org/) database^26^ was used to explore interactions among the DEGs in the protein‐protein interaction (PPI) network. An interaction with a combined score >0.4 was regarded as statistically significant.

## 3 RESULTS

### 3.1 Differential expression analysis of NEK8

**FIGURE 1:**
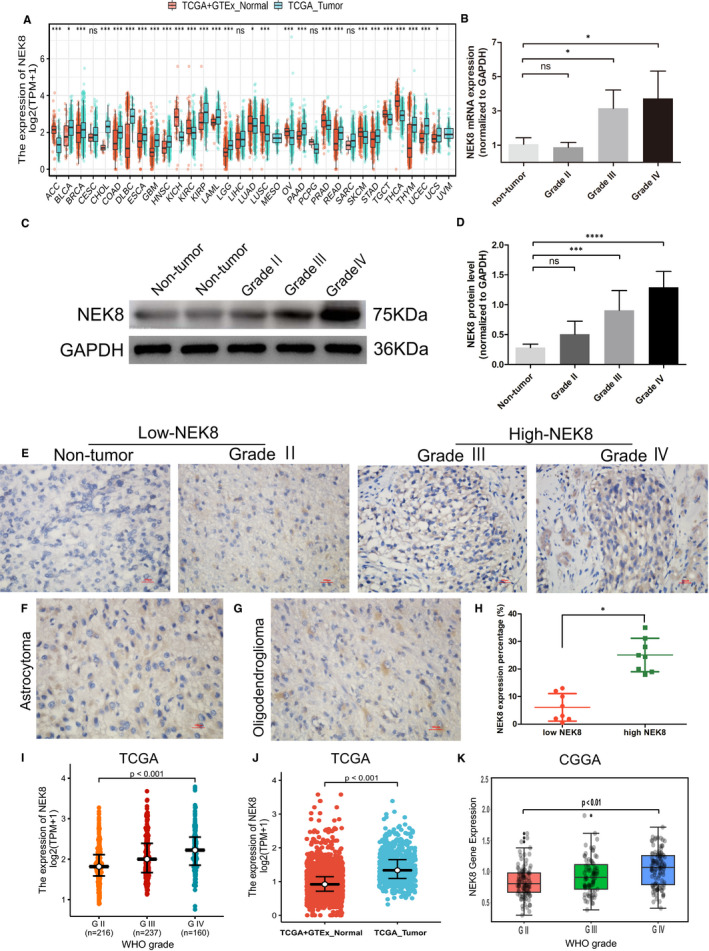
NEK8 expression in a pan‐cancer database and clinical samples. (A) Comparison of NEK8 expression between cancer tissues and control samples in 33 tumour types. (B) Bar charts of normalized mRNA expression levels of NEK8 in glioma and non‐tumour samples. (C) Representative Western blotting images of NEK8 in glioma and non‐tumour samples. (D) Bar charts of normalized protein expression levels of NEK8 in glioma and non‐tumour samples. (E–G) Representative IHC staining images of NEK8 in WHO grades, astrocytoma and oligodendroglioma. (H) Semi‐quantitative expression analysis of NEK8 in the low and high NEK8 group. The non‐tumour and grade II were set as the low NEK8 group, while grades III and IV were set as the high NEK8 group. (I–J) Analysis of the differential expression of NEK8 between glioma samples and normal samples in TCGA datasets. (K) Analysis of the differential expression of NEK8 with WHO grade between glioma and normal samples in CGGA. Data have been presented as normalized mean ± SD. Ns: *p* ≥ 0.05; *: *p* < 0.05; **: *p* < 0.01; * * *: *p* < 0.001. ACC, adrenocortical carcinoma; BLCA, bladder urothelial carcinoma; BRCA, invasive breast carcinoma; CHOL, cholangiocarcinoma; COAD, colon adenocarcinoma; DLBC, Lymphoid Neoplasm Diffuse Large B‐cell Lymphoma; ESCA, oesophageal carcinoma; GBM, glioblastoma multiforme; HNSC, Head and Neck squamous cell carcinoma; KICH, kidney chromophobe; KIRC, Kidney renal clear cell carcinoma; KIRP, Kidney renal papillary cell carcinoma; LAML, acute myeloid leukaemia; LGG, brain lower grade glioma; LUAD, Lung adenocarcinoma; LUSC, Lung squamous cell carcinoma; OV, Ovarian serous cystadenocarcinoma; PAAD, Pancreatic adenocarcinoma; PRAD, Prostate adenocarcinoma; READ, Rectum adenocarcinoma; SKCM, Skin Cutaneous Melanoma; STAD, Stomach adenocarcinoma; TGCT, Testicular Germ Cell Tumours; THCA, Thyroid carcinoma; THYM, Thymoma; UCEC, Uterine Corpus Endometrial Carcinoma; UCS, Uterine Carcinosarcoma

We first analysed the pan‐cancer expression of NEK8 by using the combined data gathered from TCGA and GTEx. As determined by the Wilcoxon rank‐sum test, NEK8 expression was upregulated in the majority of cancer types, including glioblastoma and low‐grade glioma, compared with normal tissues (Figure 1A). We then performed quantitative PCR using our clinical tissue samples and found that the mRNA levels of NEK8 were higher in both grade III and grade IV samples than in non‐tumour specimens (Figure 1B). A Western blotting assay showed that NEK8 protein expression was significantly upregulated in grade III and IV glioma samples compared with non‐tumour (Figure 1C, D). IHC staining suggested that patients with high level of positive NEK8 expression corresponded to the high glioma grade (Figure 1E) and further confirmed by the ImageJ software semi‐quantitative expression analysis (Figure 1H). Moreover, we found that NEK8 was partially expressed both in astrocytoma and oligodendroglioma (Figure 1F–G). These results were consistent with those of analyses of NEK8 data in TCGA and CGGA (Figure 1I–K), which also showed a clear trend towards increased expression according to the WHO grade.

### 3.2 Correlation between the NEK8 expression level and prognosis in glioma

**FIGURE 2:**
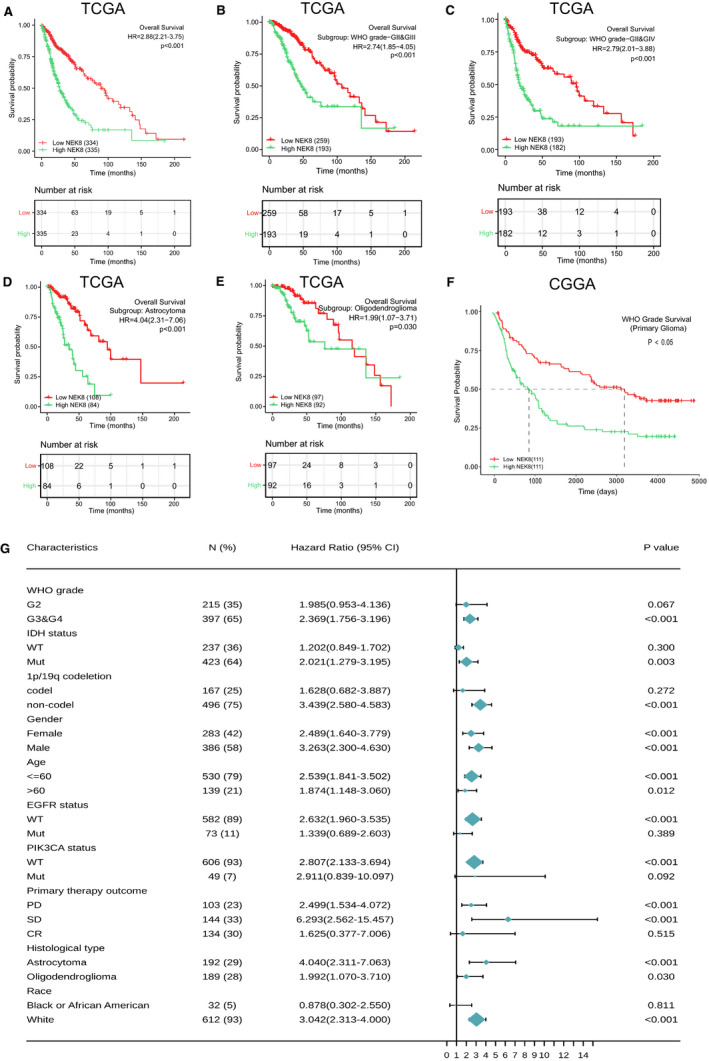
Prognostic analysis of NEK8. (A) Survival curve for NEK8 using data obtained from TCGA. The risk table recorded the number of patients followed up at various time points. (B–C) Kaplan‐Meier prognostic analysis of WHO grades III and IV compared to grade II according to the level of NEK8 expression. The risk table recorded the number of patients followed up at various time points. (D–E) Kaplan‐Meier prognostic analysis of astrocytoma and oligodendroglioma according to the level of NEK8 expression. The risk table recorded the number of patients followed up at various time points. (F) Kaplan‐Meier prognostic analysis based on the CGGA database according to the level of NEK8 expression. (G) Univariate Cox prognostic analysis of the correlation between NEK8 expression with clinical‐pathological factors. The NEK8 expression level showed significant prognostic value in the following subgroups: grade III & IV (*p* < 0.001), IDH mutation (*p* = 0.003), non‐codeletion (*p* < 0.001) for the 1p/19q codeletion, female (*p* < 0.001) and male (*p* < 0.001), ≤60 years of age (*p* < 0.001) and >60 years of age (*p* = 0.012), wild‐type (WT) EGFR status (*p* < 0.001), WT PIK3CA status (*p* < 0.001), progressive disease (*p* < 0.001) and stable disease (*p* < 0.001) for the primary therapy outcome; astrocytoma (*p* < 0.001) and oligodendroglioma (*p* = 0.03) histological types and white race (*p* < 0.001)

We then performed a Kaplan‐Meier survival analysis using data obtained from TCGA to investigate the prognostic value of NEK8 in glioma. As shown in Figure 2A–C, a worse prognosis was observed in the high NEK8 expression group than in the low NEK8 expression group when considering OS, PFI (Figure S1A) and DSS (Figure S1B) (*p* < 0.001). Similar results were obtained using data in the CGGA database (Figure 2F).

We evaluated the impact of clinical variables and NEK8 on glioma overall survival by a Cox regression analysis. In univariate analyses, the clinicopathologic variables associated with OS were WHO grade (*p* < 0.001), IDH status (*p* < 0.001), 1p/19q codeletion (*p* < 0.001), primary therapy outcome (*p* < 0.001), age (*p* < 0.001), EGFR status (*p* < 0.001), histological type (*p* = 0.005) and NEK8 (*p* < 0.001) (Table 1). To further identify factors correlated with prognosis, the clinical features with statistical significance in univariate analyses were included in a multivariate Cox regression analysis. In summary, WHO grade (*p* = 0.028), IDH status (*p* = 0.007), 1p/19q codeletion (*p* = 0.043), primary therapy outcome (*p* = 0.002), age (*p* < 0.001) and NEK8 expression (*p* < 0.001) were identified as independent prognostic factors associated with OS (Table 1).

**TABLE 1:** Univariate/multivariate Cox regression analysis of risk factors according to overall survival (OS)

Characteristics	Total (*N*)	HR (95% CI) univariate analysis	*p*‐value univariate analysis	HR (95% CI) multivariate analysis	P‐value multivariate analysis
WHO grade (G IV vs. G II &G III)	612	9.504 (7.162–12.611)	<0.001	4.099 (1.166–14.413)	0.028
IDH status (WT vs. Mut)	660	9.850 (7.428–13.061)	<0.001	2.326 (1.253–4.317)	0.007
1p/19q codeletion (codel vs. non‐codel)	663	0.216 (0.138–0.338)	<0.001	0.522 (0.278–0.979)	0.043
Primary therapy outcome (CR vs. PD & SD & PR)	443	0.238 (0.115–0.489)	<0.001	0.285 (0.130–0.629)	0.002
Gender (Male vs. Female)	669	1.230 (0.955–1.585)	0.109		
Age (> 60 vs. ≤60)	669	4.716 (3.609–6.161)	<0.001	4.116 (2.459–6.889)	< 0.001
Race (White vs. Asian & Black or African American)	657	0.806 (0.492–1.321)	0.393		
EGFR status (Mut vs. WT)	655	3.628 (2.672–4.927)	<0.001	1.810 (0.858–3.817)	0.119
PIK3CA status (Mut vs. WT)	655	1.011 (0.625–1.635)	0.966		
Histological type (Astrocytoma vs. Oligodendroglioma)	381	1.783 (1.192–2.666)	0.005	1.271 (0.681–2.373)	0.451
NEK8 (High vs. Low)	669	2.879 (2.212–3.746)	<0.001	2.633 (1.606–4.317)	< 0.001

Subsequently, as shown in Figure 2B&C, higher expression of NEK8 was correlated with a worse OS in the WHO grade III and IV subgroup (*p* < 0.001). We also discovered that higher NEK8 expression related to a poorer OS both in astrocytoma and oligodendroglioma (Figure 2D–E). Correlations between the level of NEK8 expression and major clinical features are shown in a forest plot in Figure 2D. Finally, a Kaplan‐Meier prognostic analysis suggested that high NEK8 expression is associated with a worse PFI and DSS in the different subgroups of glioma (*p* < 0.05) (Figure S1C–L). Collectively, these results showed that NEK8 is a potential prognostic marker for patients with glioma.

### 3.3 NEK8‑related prognostic nomogram

**FIGURE 3:**
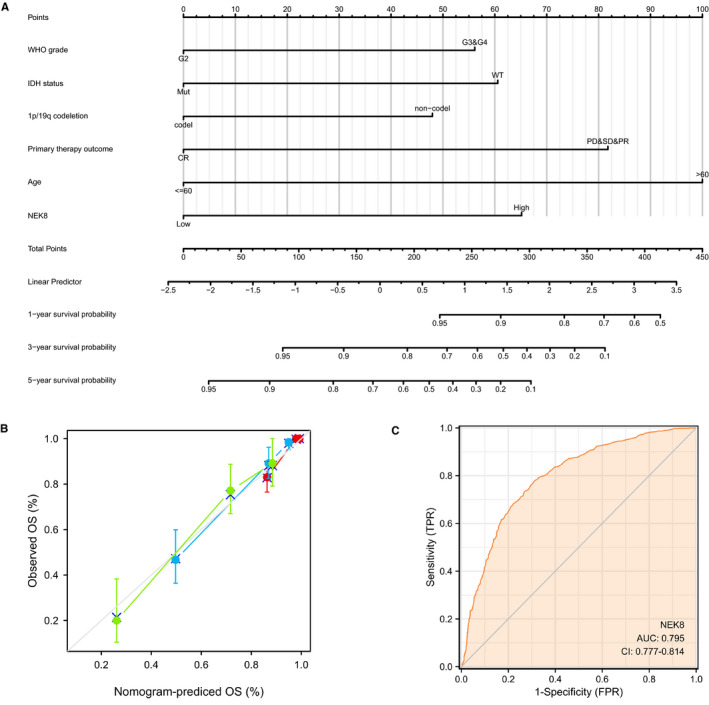
NEK8‐related prognostic model. (A) Calibration curve for NEK8. The abscissa is the probability of nomogram‐predicted OS, and the ordinate is the observed OS. (B) Nomogram for 1‐, 3‐, and 5‐year survival. The C‐index is generally between 0.5–1, where a value of 0.50–0.70 indicates low accuracy, 0.71–0.90 indicates moderate accuracy, and >0.90 indicates high accuracy. (C) ROC curve showing that NEK8 effectively discriminates glioma from normal tissues. The abscissa is the false‐positive rate, and the ordinate is the true‐positive rate

A nomogram was established to integrate NEK8 and other independent prognostic factors identified in the multivariate Cox regression analysis, including the WHO grade, IDH status, 1p/19q codeletion and age. A higher score based on the nomogram indicated a worse prognosis, and survival periods of 1, 3 and 5 years were evaluated. The C‐index value for the prediction model was 0.867, indicating a moderate predictive accuracy for OS in glioma (Figure 3A). To verify the predictive value, we used variables included in the nomogram to construct a calibration curve. The bias‐corrected curve in the calibration plot conformed well to the ideal line (the 45° line), demonstrating an excellent predictive ability (Figure 3B). The corresponding ROC curve for NEK8 expression is shown in Figure 3C. The area under the curve (AUC) was 0.795, indicating good performance. These data demonstrated that the nomogram could be used to accurately predict 1‐, 3‐ and 5‐year survival in patients with glioma.

### 3.4 Correlations between NEK8 expression levels and clinicopathologic characteristics

**FIGURE 4:**
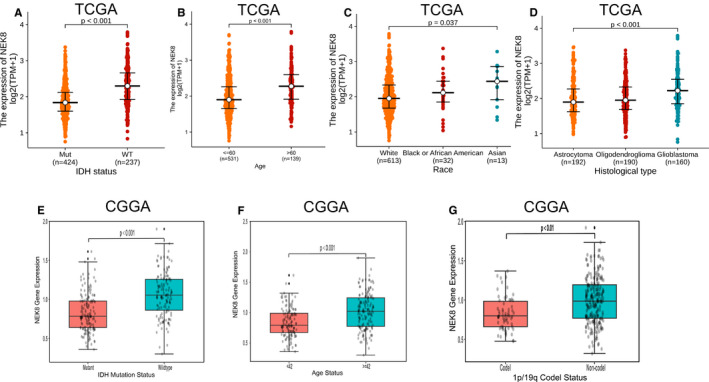
Association between NEK8 expression and clinicopathological factors. Relationships between NEK8 expression and (A) IDH status, (B) age, (C) race, (D) histological type, (E) IDH mutation status, (F) age status and (G) 1p/19q codeletion status

In an evaluation of correlations between NEK8 expression and clinicopathologic variables, we found that NEK8 expression was higher in patients with WT IDH than those with mutant IDH (*p* < 0.001) (Figure 4A). We also found that the expression level of NEK8 was higher in older patients (>60 years) and lower in younger patients (≤60 years) (*p* < 0.001) (Figure 4B). As shown in Figure 4C, the expression level of NEK8 was significantly higher in Asian and black or African American patients with glioma than that observed with white (*p* = 0.037). The expression level of NEK8 was significantly higher in glioblastoma than in other histological types (*p* < 0.001; Kruskal‐Wallis rank‐sum test) (Figure 4D). Similar results were observed using data from the CGGA database (Figure 4E–G). Moreover, the expression level of NEK8 was higher in patients with the 1p/19q non‐codeletion status than those with the codeletion status (*p* < 0.01), based on the CGGA datasets (Figure 4G). To further demonstrate the clinical significance of NEK8 protein expression, we analysed the relationship between clinical characteristics and the groups with low and high NEK8 expression. As presented in Table 2, in the high expression group, grade IV was more frequent type compared with low‐expression group (*p* < 0.01). The majority of cases in the low‐expression group showed mutant IDH (63%), while an opposite trend of IDH statuses was found in the high expression group (*p* < 0.001). Complete response (CR) and stable disease (SD) was more common primary therapy outcome in the low‐expression group (*p* = 0.034). Asian expressed high level of NEK8 more easily in glioma, while a nearly equal distribution of NEK8 was found in White (*p* = 0.017). We found that the older over 60 years old (70.6%) were more likely to express high level of NEK8 (*p* < 0.001). In the high expression group, glioblastoma was most frequent, and a similar distribution of astrocytoma and oligodendroglioma was discovered both in the low and high NEK8 expression group (*p* < 0.001). High NEK8 expression group had a worse prognosis in OS event (60.7%) (*p* < 0.001).

**TABLE 2:** Correlation between the clinical variables in TCGA and NEK8 high‐ and low‐expression groups

Characteristic	Levels	Low expression of NEK8	High expression of NEK8	*p*
n		348	348	
WHO grade, *n* (%)	G IV	56 (33.3%)	112 (66.7%)	<0.01
IDH status, *n* (%)	WT	66 (26.8%)	180 (73.2%)	<0.001
	Mut	277 (63%)	163 (37%)	
1p/19q codeletion, *n* (%)	codel	89 (52%)	82 (48%)	0.612
	non‐codel	256 (49.4%)	262 (50.6%)	
Primary therapy outcome, *n* (%)	PD	54 (48.2%)	58 (51.8%)	0.034
	SD	92 (62.6%)	55 (37.4%)	
	PR	31 (48.4%)	33 (51.6%)	
	CR	86 (61.9%)	53 (38.1%)	
Gender, *n* (%)	Female	156 (52.3%)	142 (47.7%)	0.319
	Male	192 (48.2%)	206 (51.8%)	
Race, *n* (%)	Asian	3 (23.1%)	10 (76.9%)	0.017
	Black or African American	11 (33.3%)	22 (66.7%)	
	White	329 (51.6%)	308 (48.4%)	
Age, *n* (%)	<=60	306 (55.3%)	247 (44.7%)	<0.001
	>60	42 (29.4%)	101 (70.6%)	
Histological type, *n* (%)	Astrocytoma	107 (54.9%)	88 (45.1%)	<0.001
	Glioblastoma	56 (33.3%)	112 (66.7%)	
	Oligodendroglioma	103 (51.8%)	96 (48.2%)	
OS event, *n* (%)	Alive	241 (56.8%)	183 (43.2%)	<0.001
	Dead	107 (39.3%)	165 (60.7%)	
Age, median (IQR)		39 (32, 53)	52 (39, 63)	<0.001

Note

Chi‐square tests and Fisher's exact tests were used to evaluate WHO grade, IDH status, primary outcome, histological type and OS event. The *t* test or Wilcoxon rank‐sum test was used to evaluate age.

Similarly, univariate analyses using logistic regression indicated that NEK8 expression is correlated with clinicopathologic characteristics (Table 3). NEK8 expression in glioma was significantly associated with the WHO grade (OR = 3.05 for Grade IV vs. II and III), IDH status (OR = 4.60 for WT vs. Mutant), EGFR status (OR = 4.07 for WT vs. Mutant), PIK3CA status (OR = 2.17 for WT vs. Mutant), primary therapy outcome (OR = 0.58 for CR vs. PD, SD and PR), age (OR = 0.31 for ≤60 years vs. >60 years), and histological type (OR = 2.91 for astrocytoma vs. glioblastoma). These findings suggested that NEK8 is closely correlated with clinicopathologic factors mentioned above in glioma.

**TABLE 3:** Relationship between clinicopathological features of glioma and NEK8 high or low expression, as analysed using logistic regression

Characteristics	Odds ratio for NEK8 expression	Odds ratio (OR)	*p*‐value
WHO grade (Grade IV vs. II & III)	613	3.05 (2.09–4.51)	<0.001
IDH status (WT vs. Mut)	661	4.60 (3.26–6.54)	<0.001
1p/19q codeletion (codel vs. non‐codel)	664	0.97 (0.68–1.37)	0.858
Primary therapy outcome (CR vs. PD&SD&PR)	444	0.58 (0.38–0.89)	0.012
EGFR status (Mut vs. WT)	656	4.07 (2.34–7.48)	<0.001
PIK3CA status (Mut vs. WT)	656	2.17 (1.19–4.12)	0.014
Histological type (Astrocytoma vs. Glioblastoma)	352	2.91 (1.88–4.55)	<0.001
Gender (Female vs. Male)	670	0.95 (0.70–1.29)	0.755
Race (Asian vs. White & Black or African American)	658	2.29 (0.74–8.50)	0.173
Age (≤60 years vs. >60 years)	670	0.31 (0.20–0.46)	<0.001

### 3.5 Differentially expressed genes between the NEK8 high and low‐expression groups and functional enrichment

**FIGURE 5:**
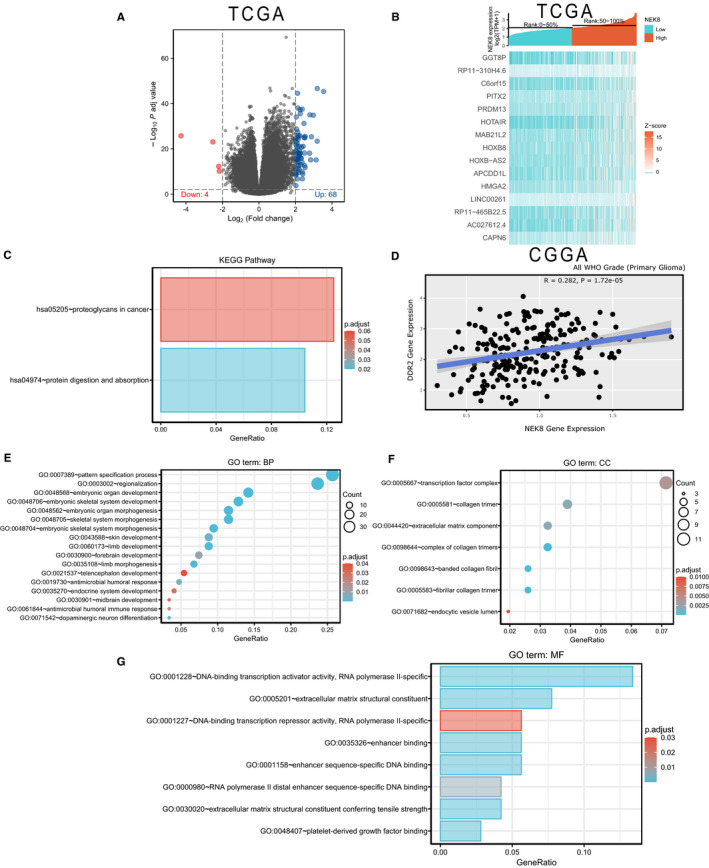
Differential expression analysis of NEK8 high‐ and low‐expression groups. (A) Volcano plot. (B) Heat map showing the co‐expression of differentially expressed genes in NEK8 high‐ and low‐expression groups. (C) Results of a KEGG enrichment analysis. (D) Relationship between NEK8 and DDR2 gene expression in the WHO grade primary glioma. (E) GO terms related to biological processes (BPs) are shown in a bubble chart. (F) GO terms associated with cellular components (CCs) are shown in a bubble chart. (G) GO terms related to molecular functions (MFs) are shown in the histogram

We analysed DEGs between the groups with low and high NEK8 expression, using the median expression level as the cut‐off value. By using the criteria |log fold change (FC)| >2 and adjusted *p* < 0.01, we obtained 72 DEGs (68 upregulated and 4 downregulated) (Figure 5A). According to the log FC values, information for the top 15 genes is shown in a heat map (Figure 5B). A KEGG enrichment analysis indicated that DEGs were involved in pathways related to protein digestion and absorption and proteoglycan metabolism in the tumour microenvironment (Figure 5C). Moreover, the NEK8 and DDR2 expression levels were highly correlated for all WHO grades (Figure 5D). In the biological process category, 52 enriched GO terms were identified, mainly related to the development and morphogenesis of the organ, limb, skin, skeletal system and brain, as well as with dopaminergic neuron differentiation and the humoral immune response (Figure 5E). In the cellular component category, we detected 7 significantly enriched GO terms linked to collagen trimer, extracellular matrix component and endocytic vesicle lumen (Figure 5F). Finally, 8 enriched GO terms were detected in the molecular function category, including DNA binding and RNA polymerase II (Figure 5G). Based on these analyses, NEK8 may influence the glioma microenvironment via the DNA damage response (DDR) pathway.

### 3.6 Effects of NEK8 on infiltrating immune cells and related genes in glioma

**FIGURE 6:**
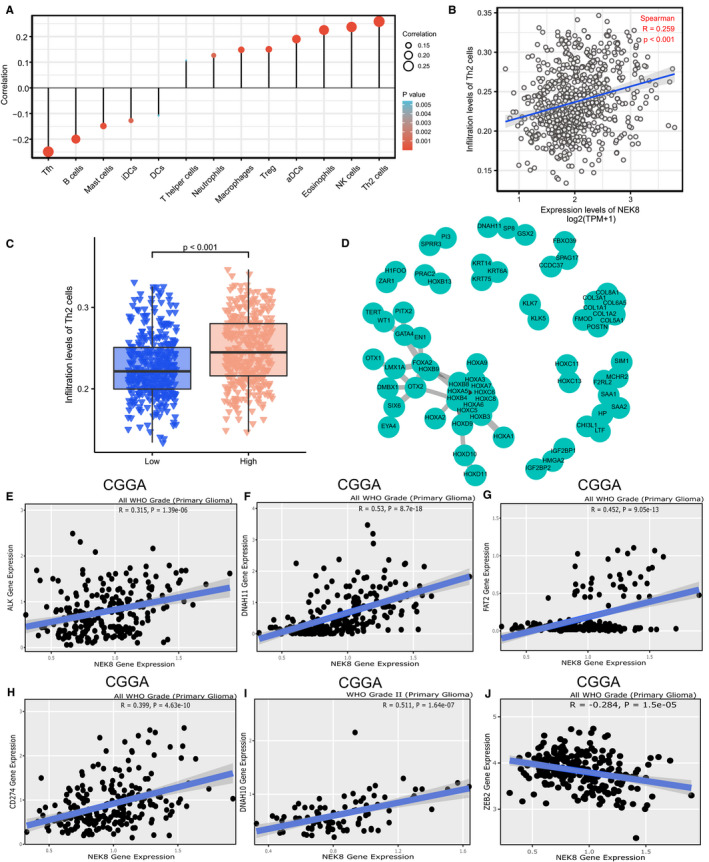
Correlation between NEK8 and immune cell infiltration. (A) Associations between the expression level of NEK8 and immune cell infiltration levels were analysed using a lollipop plot. (B) Correlations between NEK8 expression and Th2 cells infiltration. (C) Comparison of Th2 cells infiltration between the NEK8 high‐ and low‐expression groups. (D) PPI network. (E–J) Correlations between NEK8 expression and immune infiltration‐related genes in WHO grade primary glioma

**FIGURE 7:**
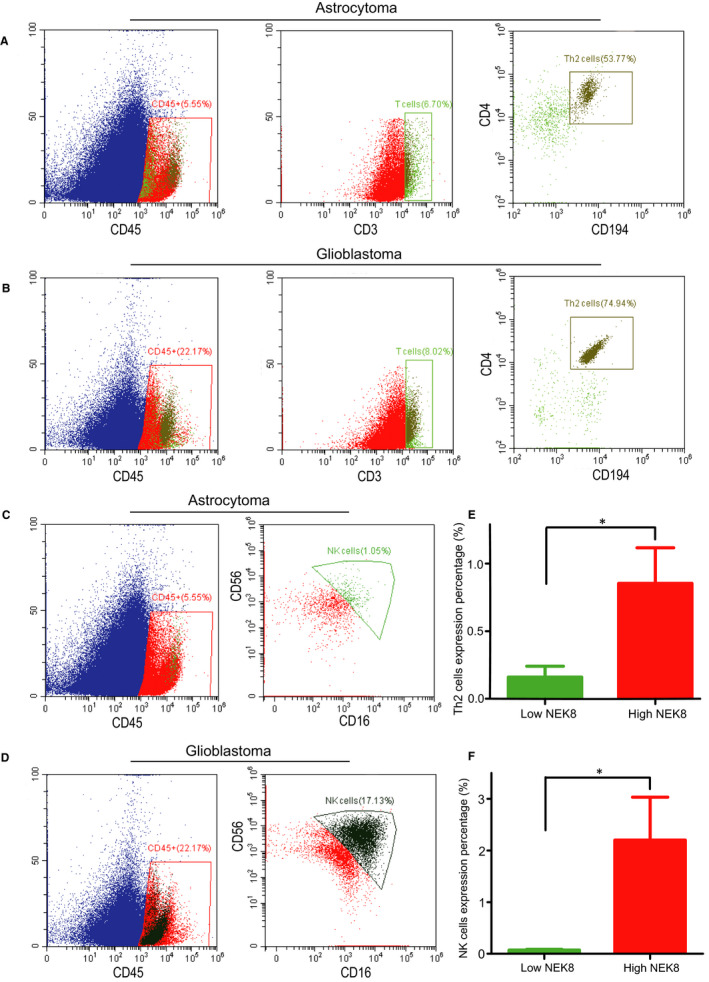
Differential expression percentage of immune cells in the high and low NEK8 expression gliomas. (A–B) Th2 cells in astrocytoma and glioblastoma were analysed by flow cytometry. (C–D) NK cells in astrocytoma and glioblastoma were detected by flow cytometry. (E–F) Bar charts of Th2 cells and NK cells expression percentage of NEK8 high‐ and low‐expression groups

We determined Spearman correlation coefficients to analyse associations between NEK8 expression and immune cell infiltration via ssGSEA in the tumour microenvironment. NEK8 expression was positively associated with the abundances of Th2 cells, NK cells, eosinophils and other cell types and was negatively associated with the abundances of T follicular helper cells, B cells and mast cells (Figure 6A, also see Figure S2A–K). The correlation between NEK8 expression and Th2 cell infiltration was significant (Figure 6B; *R* = 0.259, *p* < 0.001). In a comparison between the high‐ and low‐expression groups, we found that the high NEK8 expression indicates a significantly higher level of Th2 cell infiltration (Wilcoxon rank‐sum test; Figure 6C). Additionally, Th17 cell, regulatory T cell, T gamma delta cell, Tfh cell, T central memory cell, T helper cell, NK CD56dim cell, neutrophil, mast cell, macrophage, eosinophil, dendritic cell (DC), activated DC and B‐cell infiltration differed significantly between the NEK8 high‐ and low‐expression groups (Figure S2L–Y). Finally, a PPI network analysis of DEGs illustrated that these genes are closely correlated with biomorph regulation and development (Figure 6D). Interestingly, mutations in immune infiltration‐related genes^27^ were significantly associated with NEK8 expression in glioma datasets from CGGA. These genes included *DNAH10*, *DNAH11*, *ALK*, *FAT2*, *ZEB2* and *CD274* (Figure 6E–J). We performed flow cytometry to further confirm the above speculation using our clinical tissue samples. We calculated that the expression percentage of Th2 cells in glioblastoma (Grade IV) was about 1.33% (Figure 7B), while little was expressed in the astrocytoma (Grade II) (Figure 7A). Similarly, we detected that the expression percentage of NK cells in glioblastoma was approximately 3.8% (Figure 7D), and little was expressed in the astrocytoma (Figure 7C). As shown in Figure 7E–F, there were significant differences in expression of NK cells and Th2 cells in the high and low NEK8 expression gliomas. The above trends were consistent with recent research^28^ and revealed that NEK8 affects the infiltration of immune cells in the glioma microenvironment.

## 4 DISCUSSION

NEK8 plays an essential role in cell cycle regulation from the G2 to M phase and encodes a serine/threonine‐specific protein kinase.^6^, ^8^ These two functions may explain the role of NEK8 in the occurrence and development of cancer. NEK8 is overexpressed in breast and pancreatic cancer and affects prognosis.^22^, ^23^ However, the expression of NEK8 and its clinical prognostic value on glioma has not been investigated. In the present study, we performed bioinformatics analyses of high‐throughput RNA sequencing data. We found that elevated *NEK8* expression in glioma is associated with various clinical and pathological parameters (WHO grade, histological type, IDH, EGFR, PIK3CA status and primary therapy outcome) and survival time. A functional enrichment analysis of TCGA data further showed that the high NEK8 expression group was enriched for terms related to development and morphogenesis of organs, tissues, proliferation, differentiation and the tumour microenvironment. Additionally, elevated NEK8 expression was associated with levels of immune cell infiltration. These findings indicated that NEK8 might serve as a potential prognostic marker and therapeutic target in glioma.

We developed novel nomograms, showing better performance than that of standard staging systems.^29^, ^30^ The nomogram included eleven parameters from clinical records and tissue samples. As previously reported, age is an independent prognostic factor and is positively correlated with a poor prognosis.^31^ PIK3CA and IDH mutations are an early event in glioma and are associated with progression.^32^, ^33^ WT PIK3CA, IDH and high WHO grade (III or IV) may be associated with poor outcomes, while the opposite results have been obtained for EGFR.^34^ With respect to gender, rates of tumorigenesis are higher in males than in females.^35^ Astrocytoma is a common malignant glioma with a poor prognosis.^36^ These results are consistent with those of our study. The C‐index values, AUC values and calibration plots suggested that the nomogram effectively predicts 3‐ or 5‐year survival for patients with glioma; accordingly, the nomogram may be a valuable clinical tool for patients with glioma.

Our GO enrichment analysis suggested that NEK8 is strongly associated with DNA binding, RNA polymerase II and extracellular matrix components; it was also related to the development and morphogenesis of the limbs, skin, skeletal system, brain and organs. NEK8 might promote the development of glioma via DNA damage/repair.^37^ Additionally, a KEGG functional analysis suggested that NEK8 is involved in the microenvironment of glioma. Previous studies have shown that NEK2, NEK4, NEK8, NEK10 and NEK11 are related to genome instability and mutations.^38^, ^39^ Similarly, NEK8 is a critical regulator of replication and proliferation; the deletion of NEK8 results in DNA double‐strand breaks in the S phase and the accumulation of DNA damage.^21^ DDR plays a crucial role in maintaining genome stability; DDR alterations increase the risk of tumour occurrence and development.^40^ The tumour microenvironment refers to the interactions between cancer cells and their surrounding cells, such as cancer‐associated fibroblasts, throughout the stages of cancer progression, leading to a poor prognosis.^41^ DNA damage affects the tumour microenvironment via a range of molecular and cellular mechanisms; for example, it decreases genomic stability, activates immune pathways and upregulates programmed death‐ligand 1 (PD‐L1) expression, which increases the complexity of cancer treatment.^42^ Clinical oncology has made significant breakthroughs in the development of therapies targeting DNA repair.^43^ For instance, the key transcription factor p53 in the DDR pathway can affect the glioma microenvironment in immunotherapy.^44^ Other DDR targets, such as DNA‐PKcs, ATM/ATR, DNA LIG4, HDAC, and CDK1, have also been identified.^45^ Despite these advances, primary or acquired resistance often results in tumour escape.^46^

The biological toxicity and mechanisms of action of inhibitors of DDR are not completely understood; this emphasizes the importance of statistical approaches for the exploration of accurate and predictive biomarkers based on large datasets.^45^, ^46^ Our bioinformatics analyses revealed that NEK8 is closely correlated with DDR in glioma. In addition, we elucidated that NEK8 might modulate the glioma microenvironment via the DDR pathway and, therefore, is a target for suppressing DDR in glioma.

Furthermore, DDR contributes to the immune composition of the tumour microenvironment in glioma.^44^ In particular, DDR alterations are related to immunosuppression and to the positive regulation of cytokine biosynthesis.^44^ Tumours with DDR alterations avoid host immune‐mediated elimination by activating immunosuppression.^47^ The changes in the immune microenvironment result in the release of excessive cytokines and chemokines to coordinate immune responses, leading to the infiltration of various immune cells affecting tumour behaviour and prognosis.^48^ We also detected that NEK8 affects the infiltration of immune cells in the glioma microenvironment. High NEK8 expression was associated with a high percentage of activated dendritic cells, plasmacytoid dendritic cells, macrophages, NK cells and Th2 cells. In glioma, immune cells aggregate and are modified to escape the host immune system surveillance.^49^ For instance, glioma cells induce the abnormal expression of Nrf2 in DCs to suppress their maturation and T‐cell activation, finally leading to immune escape.^50^ Domingues et al. observed that DCs downregulate costimulatory molecules (CD40, B7.1 and B7.2) and fail to stimulate T cells in a mouse model of glioma.^51^

Additionally, glioma cells positively recruit microglia/macrophages and induce M2 polarization.^52^ Interestingly, glioma‐associated M2 macrophages are more highly distributed in the DDR cluster2 tissues.^44^ M2 microglia are also differentially expressed in glioma samples with DDR alterations.^44^ Moreover, M2‐polarized macrophage infiltration is associated with a poor prognosis in high‐grade gliomas and with an aggressive glioma subtype.^53^ These changes generate a supportive environment, promote a variety of immune responses and maintain glioma growth and progression.^54^ Therefore, it is necessary to explore the mechanism by which NEK8 influences the infiltration of immune cells in the glioma microenvironment, in future research.

Although our results improve our understanding of the relationship between NEK8 and the pathogenesis of glioma, the study had some limitations. First, cell or animal experiments were not performed. We used a bioinformatics approach based largely on RNA sequencing data from TCGA and CGGA. Second, the research was performed at multiple institutions, which can lead to gaps in data processing and analysis via inconsistent methods. Third, although multi‐centre studies can address various drawbacks of single‐centre studies, retrospective studies still have important limitations. Therefore, additional prospective studies are needed to avoid analysis bias. Broadly, further analyses of the precise role of NEK8 in glioma are needed.

NEK8 participates in cell cycle regulation^6^, ^8^ and the maintenance of replication stability via the regulation of DNA repair and the replication protein RAD51.^37^ We further showed that NEK8 expression is elevated in glioma and is associated with the WHO grade. High NEK8 expression is associated with a poor survival. A nomogram including NEK8 was established to precisely predict 1‐, 3‐ and 5‐year survival for patients with glioma. The effects of NEK8 in glioma may be mediated by alterations in immune cell infiltration into the tumour microenvironment via the regulation of DDR. These results provide insights into the biological properties of glioma and may facilitate the development of molecular markers to effectively assess prognosis, improve treatment and accelerate drug development.

## 5 CONCLUSIONS

We elucidated that NEK8 expression is increased in glioma and is associated with the WHO grade and prognosis. We established a nomogram including NEK8 to effectively predict 1‐, 3‐ and 5‐year year survival for patients with glioma. With respect to biological functions, we elucidated that NEK8 influences immune cell infiltration into the glioma microenvironment via the regulation of DDR. These results suggest NEK8 may serve as a prognostic biomarker and therapeutic target for glioma.

## CONFLICT OF INTEREST

The authors declare that the research was conducted in the absence of any commercial or financial relationships that could be construed as a potential conflict of interest.

## AUTHOR CONTRIBUTIONS

**Meng Xiao:** Data curation (equal); Project administration (equal); Writing‐original draft (equal). **Chaoyang Du:** Project administration (equal). **Chuanbo Zhang:** Software (equal). **Xinzhong Zhang:** Conceptualization (equal); Resources (equal). **Shaomin Li:** Formal analysis (supporting); Resources (supporting). **Dainan Zhang:** Conceptualization (supporting); Funding acquisition (supporting); Writing‐review & editing (supporting). **Wang Jia:** Funding acquisition (lead); Supervision (lead); Writing‐review & editing (lead).

## Supporting information

Fig S1

Fig S2

Table S1
