# Supplementary figures and images for: Bioinformatics analysis of the prognostic value of NEK8 and its effects on immune cell infiltration in glioma
Source: J Cell Mol Med. 2021 Aug 10;25(18):8748–63. doi: 10.1111/jcmm.16831 (PMC8435421; doi:10.1111/jcmm.16831)

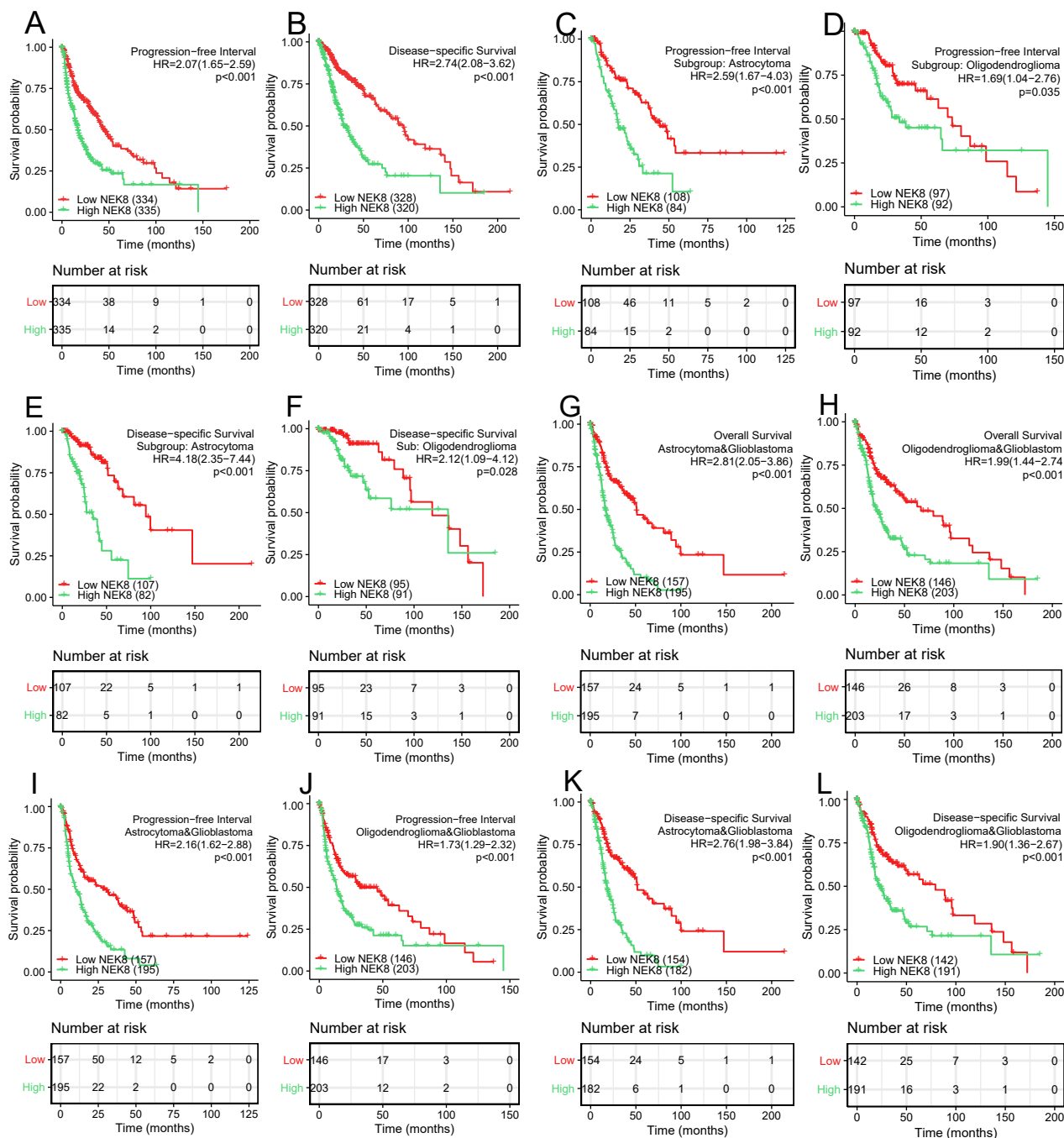

Supplement: Supplementary file 1 — Fig S1 [file JCMM-25-8748-s003.pdf]

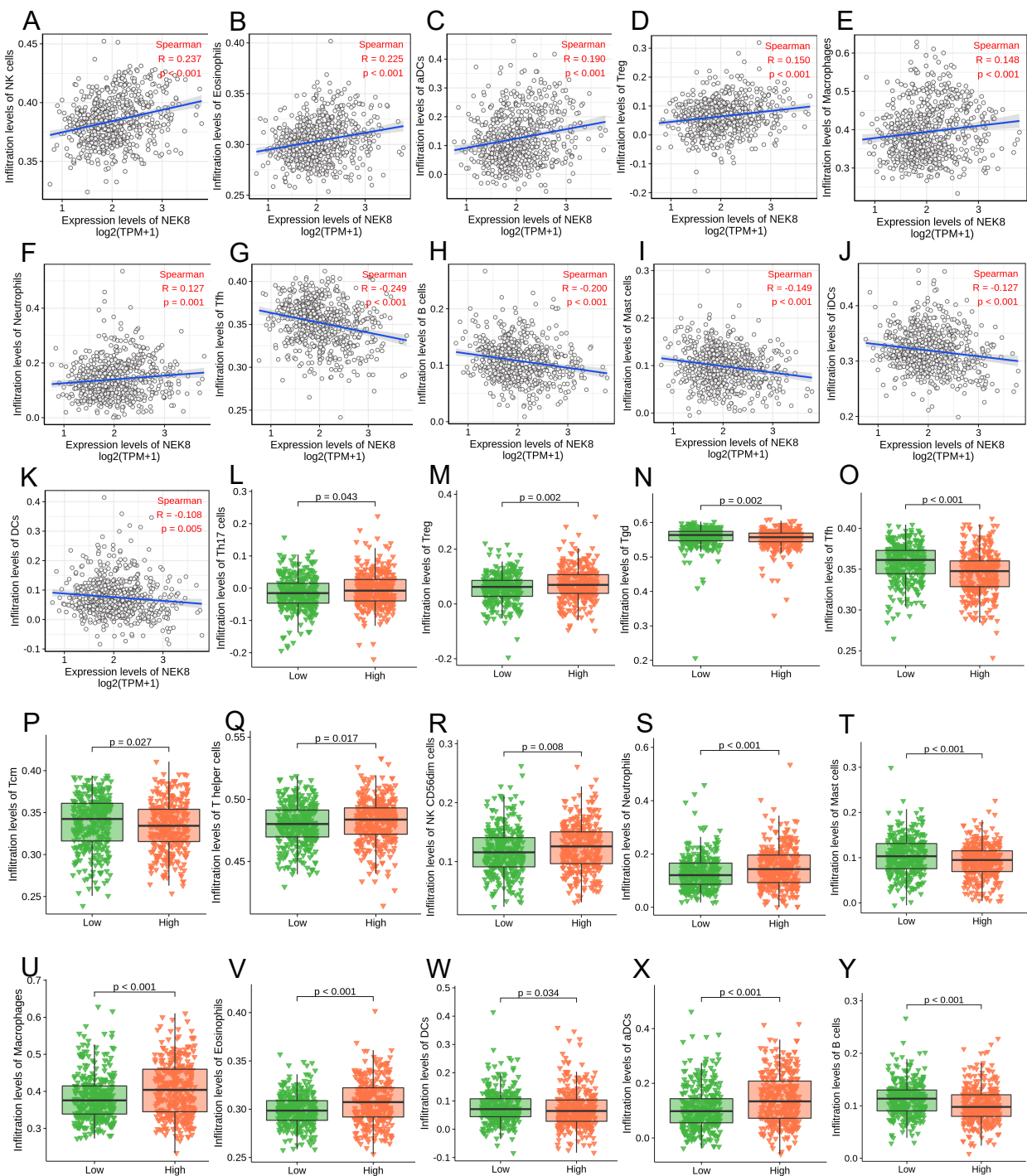

Supplement: Supplementary file 2 — Fig S2 [file JCMM-25-8748-s002.pdf]
