# Supplementary material for: Bioinformatics analysis of the prognostic value of NEK8 and its effects on immune cell infiltration in glioma
Source: J Cell Mol Med. 2021 Aug 10;25(18):8748–63. doi: 10.1111/jcmm.16831 (PMC8435421; doi:10.1111/jcmm.16831)
Supplement: Supplementary file 3 — Table S1 [file JCMM-25-8748-s001.docx]

| **Hospital number** | **Name** | **Gender** | **Age** | **Diagnose** | **WHO grade** | **IDH status** |
| --- | --- | --- | --- | --- | --- | --- |
| 5157945 | Jinnan Hu | Male | 29 | Glioblastoma | IV | MT |
| 5169089 | Jinchang Li | Male | 69 | Glioblastoma | IV | MT |
| 5169133 | Tiancai Hao | Male | 71 | Glioblastoma | IV | MT |
| 5172319 | Jumin Zhang | Male | 62 | Anaplastic astroglioma | III | MT |
| 5173450 | Wenhua Guo | Male | 47 | Glioblastoma | IV | MT |
| 5175554 | Aifeng Li | Female | 61 | Glioblastoma | IV | MT |
| 5175561 | Chongyan Wang | Male | 61 | Glioblastoma | IV | MT |
| 5178090 | Yuzhen Ding | Female | 57 | Glioblastoma | IV | MT |
| 5199717 | Qiuxi Li | Male | 69 | Glioblastoma | IV | MT |
| 5195199 | Qingquan Ma | Male | 46 | Glioblastoma | IV | MT |
| 5206986 | Jinju Nie | Male | 53 | Oligodendroglioma | II | Mut |
| 5205463 | Wenqun Jia | Male | 64 | Anaplastic astroglioma | III | MT |
| 5213111 | Changxin Yu | Female | 53 | Glioblastoma | IV | Mut |
| 5219184 | Jinhua Li | Female | 66 | Glioblastoma | IV | MT |
| 5219944 | Shejun Chan | Male | 50 | Anaplastic oligodendroglioma | III | MT |
| 5220954 | Ruizhen Hao | Female | 64 | Glioblastoma | IV | MT |
| 5226733 | Lingli Guo | Female | 50 | Glioblastoma | IV | MT |
| 5227329 | Baozhen Dong | Female | 65 | Glioblastoma | IV | MT |
| 5235834 | Chunbao Zhang | Male | 63 | Glioblastoma | IV | MT |
| 5289940 | Lixia Wang | Female | 49 | Anaplastic oligodendroglioma | III | Mut |
| 5295765 | Haiping Ma | Female | 34 | Anaplastic oligodendroglioma | III | Mut |
| 5303450 | Baoqun Guo | Male | 51 | Astroglioma | II | Mut |
| 659193 | Baozhu Chang | Male | 64 | Oligodendroglioma | II | Mut |
| 279498 | Xiaoyan Cao | Female | 49 | Anaplastic oligodendroglioma | III | MT |
| 680408 | Baogang Li | Male | 58 | Oligodendroglioma | II | Mut |
| 642496 | Xuning Zhang | Male | 33 | Astroglioma | II | Mut |
| 494778 | Zizhen Wu | Female | 43 | Astroglioma | II | Mut |
| 659176 | Xianglong Meng | Male | 56 | Astroglioma | II | Mut |
| 663669 | Wenhao Ma | Male | 28 | Anaplastic astroglioma | III | Mut |
| 673435 | Bingye Li | Female | 31 | Astroglioma | II | Mut |
| 679517 | Xiuyan Li | Female | 43 | Anaplastic astroglioma | III | MT |

**Supplementary Table 1** Patients information of clinical samples.
